# Online Gambling: A Systematic Review of Risk and Protective Factors in the Adult Population

**DOI:** 10.1007/s10899-023-10258-3

**Published:** 2023-11-14

**Authors:** Michela Ghelfi, Paola Scattola, Gilberto Giudici, Veronica Velasco

**Affiliations:** 1 Psychology Department, Università degli Studi di Milano-Bicocca, Piazza dell Ateneo Nuovo 1, 20126 Milan, Italy; 2 Società Cooperativa Sociale Piccolo Principe, 24061 Bergamo, Italy

**Keywords:** Online gambling, Review, Risk factors, Protective factors, Problem gambling, Prevention

## Abstract

In recent decades, internet gambling has seen strong growth and diffusion due to intrinsic characteristics that make it particularly attractive to players (accessibility, anonymity, variety of games). This paper aims to present the current state of knowledge of the risk and protective factors of online gambling. A literature search conducted in the PubMed, PsychInfo, and Scopus databases found 42 articles, which were included in the review. Methodological aspects and risk and protective factors were analysed cross-sectionally. The results concerning risk and protective factors were distinguished by the level of analysis: individual, relational, and contextual. Two types of comparisons were considered: online vs. offline gamblers and online nonproblematic vs. problematic gamblers. The results of the two comparisons were juxtaposed to analyse their consistency and the different associations with factors. In general, the review showed that risk factors and variables at the individual level are investigated to a greater extent, while protective factors at the relational and contextual level need more in-depth study in future research. More specifically, this review found that even if online and offline gamblers shared most risk and protective factors, there are variables that they would not have in common. These factors could be important to consider in preventive interventions aimed at online gamblers and online problematic gamblers.

## Introduction

Gambling is defined as a form of entertainment centred on the wagering of any kind of valuable object or possession on a game or event, whose outcome is predominantly random (Boyd & Bolen, 1968). Since the beginning of time across all cultures and societies, gambling has been one of the most widespread leisure activities, and that has not changed. For many people, gambling is an enjoyable activity that has no repercussions on their lives; in contrast, for others, gambling may lead to addiction (Serpelloni, 2013). Previous studies have shown that the prevalence of adult problem gamblers is between 0.12 and 5.8% worldwide (Calado & Griffiths, 2016). Moreover, gambling has grown exponentially in recent decades, and accessibility, participation and expenditures are markedly increasing, as never before (Abbott, 2020). For these reasons, problem gambling is considered a socially relevant issue. It compromises public health by negatively impacting the wellbeing of individuals, their network of relationships and society as a whole. Interventions and policies, both from the point of view of care and treatment and by preventing its spread, are necessary.

Over the past 20 years, so-called internet or online gambling has grown exponentially mainly due to technological innovation (Gainsbury et al., 2012; Kim & King, 2020). Online gambling includes all forms of gambling conducted on the internet via different devices, such as laptops, mobile phones, tablets and digital TVs (Gainsbury et al., 2013). Online gambling represents an even more challenging phenomenon than offline gambling, as it is extremely widespread and characterized by more risk that make control, prevention and intervention complicated (Gainsbury, 2012). Moreover, online gambling has specific features that make it notably advantageous compared to land-based gambling: easier accessibility, convenience (less time and no travel are required), time flexibility (available 24 h a day), higher interactivity and continuity and ensured privacy (Gainsbury, 2012; Gainsbury et al., 2013). Additional reasons that make internet gambling more attractive to gamblers are the opportunity to create profiles that can hide one’s real identity and to play alone or interact with others through instant chats and forums (Hing et al., 2014).

This phenomenon has been impacted by COVID-19. Land-based gamblers have experienced massive changes during lockdowns due to the closure of gambling venues and the suspension of sports events. The pandemic has reduced overall gambling entries but has prompted land-based players to shift to internet gambling (Hodgins & Stevens, 2021). Meanwhile, the most recent literature regarding the effects of coronavirus on online gambling report no change in online gamblers’ play but no significant increase in this mode of gambling (Brodeur et al., 2021; Hodgins & Stevens, 2021). Nonetheless, higher levels of problem gambling are reported among those who have increased their gambling, and there is a strong association with mental health problems and substance use. Given these concerns, exacerbated by the COVID-19 pandemic, the diffusion of online gambling should be carefully monitored.

To design effective interventions and policies, it is essential to know the risk and protective factors associated with a phenomenon (Coie et al., 1993). However, the literature regarding the risk and protective factors of online problem gambling is not comprehensive. Most articles have focused on identifying risk and protective factors of problem gambling, especially among offline gamblers, or without even distinguishing them from online gamblers. Furthermore, most of the work concerning risk and protective factors has addressed the adolescent population (Dickson et al., 2008; Dowling et al., 2017), while little has targeted the adult population.

The most recent review regarding the risk and protective factors of internet gambling in the adult population was published by Gainsbury (2015), and it focuses on the association between online and problem gambling by comparing internet gambling with land-based gambling. However, this is not a systematic review, and no information is given about the methodology used. Given that most gamblers are not problematic, it is be important to better understand if there are differences between gamblers who choose to gamble online, without necessarily focusing on problematic gamblers. Moreover, considering the rapidly growing rate of this phenomenon, it seems necessary to update the knowledge about it to keep up with the changes.

This paper aims to review the knowledge and evidence about the factors that influence the likelihood of being an online gambler and developing a problematic mode of gambling among the adult population. To synthesize and systematize the results regarding risk and protective factors, two types of comparisons were made: comparison of factors that distinguish offline from online gamblers and comparison of online nonproblematic gamblers with online problematic gamblers. In addition, a further comparison was carried out to highlight whether similarities or differences emerged with respect to the factors studied between the first and second comparisons.

## Methods

### Search Strategy

To investigate knowledge about the risk and protective factors of online gambling, a systematic literature search was conducted in three different academic databases: PubMed, PsychInfo, and Scopus. Analogous syntaxes were launched limited to peer-reviewed articles only. The main keyword was “*gambling*” combined with “*online, internet, interactive*” and “*risk factors, protective factors, predictors, correlates*”. For clarification, the syntax entered in PsychInfo was (ab(online) OR ab(internet) OR ab(interactive)) AND ab(gambl*) AND (ab(risk factor*) OR ab(protect factor*) OR ab(promotive factor*) OR ab(predictor*) OR ab(correlate*)). Additional relevant publications were added based on the reference lists of selected papers and consultations with some experts in the gambling field. This systematic review was conducted in accordance with the Preferred Reporting Items for Systematic reviews and Meta-Analyses (PRISMA) 2015 Checklist (Moher et al., 2016).

### Inclusion Criteria

The literature search was limited to peer-reviewed studies published in English between 2010 and 2020. The decision to investigate only this decade is to focus on the current state of knowledge of a phenomenon that, especially in recent years, is spreading significantly. The eligible articles met the following inclusion criteria in terms of Population Intervention Comparison Outcome (PICO): the reference population (P) was composed of adult online gamblers (age > 18), and risk factors and/or protective factors (I) at any level (individual and environmental) were investigated, excluding those related to biological determinants. Regarding the research outcome (O), outcomes related to all degrees of addiction, severity (nonproblematic, problematic, pathological), and risk of online gaming (low, medium, high) were included. Regarding the type of comparison (C) analysed, only articles comparing online gamblers with offline gamblers (C1) and/or online nonproblematic gamblers with online problematic gamblers (C2) were included.

#### Study Selection, Data Extraction and Analysis

Two independent evaluators screened the studies and extracted the data. The selection of articles was divided into two stages. First, studies were selected by reading the title and abstract, and those that were not relevant were excluded. Once the two researchers compared their choices, only those studies considered potentially eligible by both researchers were retained. The second phase consisted of full-text reading and application of the eligibility criteria. In cases of disagreement, the article was discussed, and a consensus was reached. After selecting the papers, the following data were extracted: aim of the study, method and type of article, sample characteristics (size, representativeness, response rate, recruitment method), tools and analysis used, control or comparison group, country, population and subpopulation, variables investigated, risk and protective factors. The extraction of population type and subpopulation concerned only sociodemographic characteristics. Data were extracted, and the narrative was synthetized by 2 authors and discussed and revised by another author. Once data extraction was performed, an initial stage of analysis was carried out. According to the main aim, each paper was categorized by the type of comparison (online vs. offline, nonproblem online versus problem online, both), the level of analysis studied (individual, relational, contextual), and the type of factors investigated (protective or risk factors). Papers were not categorized by the subpopulation of gamblers in terms of type of gambler (poker players, sport bettors, etc.) to investigate the differences between online and offline gamblers net of the influence each game type could exert. The group discussed the data, and the results were based on the consensus reached.

## Results

The review results are presented below. First, the search results and the screening process are shown. Second, there is a brief presentation of the characteristics of the included papers in terms of methodology. Third, the analysis of risk and protective factors reported in the included articles is presented. In this section, the factors associated with online gambling are analysed and subdivided according to the level of analysis (individual, relational and environmental). To synthesize and systematize the results regarding risk and protective factors, two types of comparisons were made: comparison of factors that distinguish offline gamblers from online gamblers (C1) and comparison of nonproblematic online gamblers from problematic online gamblers (C2). In addition, a further comparison was carried out to highlight whether similarities or differences emerged with respect to the factors studied between the first and second comparisons (C3). The results are systematized and presented in tables at the end of the paper, see Appendix A.

### Search Results and Flowchart

**Fig. 1:**
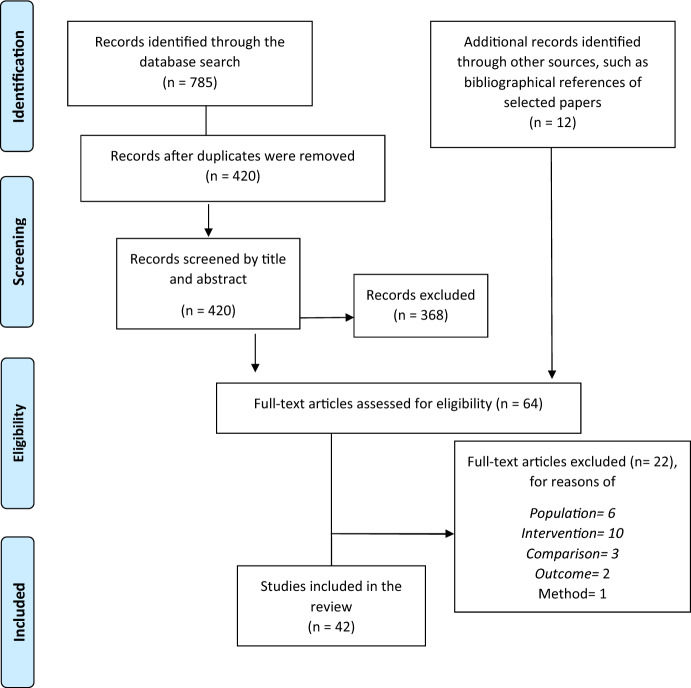
PRISMA Flow diagram

Figure 1 represents the flowchart of the screening process. In total, 785 papers were retrieved through the database search, and deleting duplicates resulted in 420 unique citations. The first step, which consisted of screening studies by title and abstract, resulted in 52 eligible articles. Furthermore, 12 studies were retrieved from reference lists and gambling experts.

The second step involved selecting studies via full-text screening in relation to PICO criteria. Of the 64 eligible articles, 42 were included in the review. Appendix B includes a table with the title, the authors, and the year of publication of the articles included in the review in chronological order from the most recent.

### Features of Selected Studies

Almost all of the selected studies were conducted in Western countries (Europe, the United Kingdom, Australia, Canada, United States of America), whereas only one was conducted in Asia (Macau) (Wu et al., 2015).

The young adult population (approximately 18–25 years) is the sample population in 8 papers) (1), most of which addressed the university population (Griffiths et al., 2010; Harris et al., 2013; Hopley & Nicki, 2010; MacKay & Hodgins, 2012; Mihaylova et al., 2013; Shead et al., 2012; Wu et al., 2015).

Quantitative methodology is used in all of the articles, mostly self-administered online questionnaires. Two studies used a mixed-method approach integrating quantitative data with semistructured interviews (Granero et al., 2020; Schiavella et al., 2018). Nearly all of the studies are cross- sectional. As these studies relate to a single measurement, the direction of the relationship between the variable and the outcome is not always clearly recognizable. Only 3 papers are longitudinal and consider different time periods: 30 days (Goldstein et al., 2016), and 2 years (Braverman & Shaffer, 2012; Dufour et al., 2020).

Some articles use random and representative samples of the population. These studies are usually part of a wider national survey. However, the sample is self-selected in most of the papers.

In most of the articles, recruitment took place on the internet, given the characteristics of the sample. The participants were recruited mostly through online advertisements on specialized websites and forums and on social networks. Another remote method commonly used concerned online wagering operators, who sent an email invitation to a randomly selected user sample. In many studies, participants were recruited using both online and offline methods, the latter including advertising in newspapers, on television, on the radio or by telephone or posters at gambling venues.

Different types of analysis were carried out for different purposes. Among the main ones are the identification of gamblers’ groups through cluster analysis (Braverman & Shaffer, 2012; Dufour et al., 2013, 2020; Granero et al., 2020; Khazaal et al., 2017; Lloyd et al., 2010a; Perrot et al., 2018) comparison between groups using bivariate or multivariate analysis, and the exploration of population characteristics through descriptive analysis.

### Risk and Protective Individual Factors

#### Sociodemographic Information

When compared to offline gamblers, online gamblers were more likely to be male male (Dowling et al., 2015; Edgren et al., 2017; Gainsbury et al., 2012; Goldstein et al., 2016; Griffiths et al., 2011; Harris et al., 2013; Kairouz et al., 2012; Lelonek-Kuleta et al., 2020; MacKay & Hodgins, 2012; Mihaylova et al., 2013; Redondo, 2015; Shead et al., 2012; Wood & Williams, 2011; Wu et al., 2015), younger (Dowling et al., 2015; Edgren et al., 2017; Gainsbury et al., 2013; Griffiths et al., 2011; Hubert & Griffiths, 2018; Kairouz et al., 2012; Lelonek-Kuleta et al., 2020; Redondo, 2015; Wardle et al., 2011; Wood & Williams, 2011; Wu et al., 2015), with a higher level of education (Dowling et al., 2015; Gainsbury et al., 2015b; Griffiths et al., 2011; Redondo, 2015; Wardle et al., 2011; Wu et al., 2015) and a higher income (Dowling et al., 2015; Edgren et al., 2017; Gainsbury et al., 2012; Wardle et al., 2011; Wood & Williams, 2011; Wu et al., 2015). Among the articles included in the review, there is a strong homogeneity of results for these four factors. The only exception is in the article by Lelonek-Kuleta et al. (2020), in which a lower income was most likely associated with internet gamblers.

In addition to these widely studied factors, further sociodemographic factors are investigated to a lesser extent. For example, regarding gamblers’ occupation, it is shown that having paid employment (Dowling et al., 2015; Wardle et al., 2011) and a full-time job (Edgren et al., 2017; Gainsbury et al., 2012, 2013; Wood & Williams, 2011) is more likely reported by internet gamblers than land-based gamblers, although Hubert and Griffiths (2018) (26) report contradictory results. Contrasting results are also reported regarding gamblers’ marital status or relationships. According to 3 articles, online gamblers are more likely to live with a stable partner (Dowling et al., 2015) or to be married (Hubert & Griffiths, 2018; Wood & Williams, 2011), whereas other studies report that they are less likely to be married Hubert & Griffiths, 2018; Wood & Williams, 2011) and more likely to be single (Griffiths et al., 2011; Kairouz et al., 2012) or cohabiting (Kairouz et al., 2012). The place of residence was investigated by two different authors, who came to opposite conclusions. According to Lelonek-Kuleta et al. (2020), living in rural areas (rather than a city or town) increases the likelihood of being an online gambler. In contrast, according to Gainsbury et al., (2015a, 2015c), internet gamblers are more likely to live in a metropolis. Another variables have been investigated: having dependent children, which is associated with both online and offline gambling (Dowling et al., 2015; Hubert & Griffiths, 2018).

The comparison between online problematic and nonproblematic gamblers shows partially different results. It was found that online problem gamblers are more likely to be male (Gainsbury et al., 2014b; Hing et al., 2017; McCormack et al., 2013b; Wu et al., 2015), younger (Gainsbury et al., 2013, 2014c, 2015c; Granero et al., 2020; Hing et al., 2017), less educated educated (Gainsbury et al., 2015c; Schiavella et al., 2018), have a lower income (Granero et al., 2020; Hing et al., 2017), be unemployed or rarely professionally active (Barrault et al., 2017; Gainsbury et al., 2014c, 2015c; Granero et al., 2020), unmarried (Gainsbury et al., 2015c; Granero et al., 2020; Khazaal et al., 2017) and have dependent children (Lelonek-Kuleta et al., 2020), than online nonproblematic gamblers.

A few articles have reported opposite results. Regarding gambler’s sex, Gainsbury et al. (2014c) reported that chasing losses, a behaviour associated with pathological gambling, is more frequent among women than men. An interesting result emerges from the comparative study by Edgren et al. (2017) in which female online gamblers were found to be at higher risk than men, both of higher expenditures on gambling and of being more problematic gamblers. Furthermore, in Khazaal et al. (2017), the higher percentage of women was within the most problematic cluster. The latter study is also in contrast with the majority of articles about the age variable, reporting that the most problematic cluster is characterized by a higher age average compared to the less problematic clusters.

### Gambling Patterns and Behaviours

Regarding gambling behaviour, differences were found between online and offline gamblers, and two variables were particularly salient: intensity and variability of gambling. Compared to land-based gamblers, internet gamblers were more likely to gamble more frequently (high intensity) (Barrault & Varescon, 2016; Dowling et al., 2015; Dufour et al., 2013; Gainsbury et al., 2012, 2013; Hubert & Griffiths, 2018; Kairouz et al., 2012; MacKay & Hodgins, 2012; Mihaylova et al., 2013; Shead et al., 2012). Consistent results from different studies state that high variability in gambling activities is associated more with online gambling than offline gambling (Dowling et al., 2015; Edgren et al., 2017; Gainsbury et al., 2012, 2013; Kairouz et al., 2012; MacKay & Hodgins, 2012; Mihaylova et al., 2013; Shead et al., 2012; Wardle et al., 2011; Wood & Williams, 2011). Furthermore, online gamblers are more likely to gamble for longer periods of time and to report higher expenditures than offline gamblers (Dowling et al., 2015; Dufour et al., 2013; Goldstein et al., 2016; Kairouz et al., 2012; Wood & Williams, 2011), as well as higher indebtedness (Mihaylova et al., 2013; Wood & Williams, 2011). In contrast with these results, Barrault and Varescon (2016) state that longer sessions, higher bets and winnings are more likely reported by offline gamblers than online gamblers.

In addition to higher intensity, variability, and expenditures, online gamblers are more likely to be at risk of problem gambling gambling (Dufour et al., 2013, 2020; Goldstein et al., 2016; Griffiths et al., 2011; Harris et al., 2013; MacKay & Hodgins, 2012; Wardle et al., 2011; Wood & Williams, 2011; Wu et al., 2015). In fact, internet gamblers have higher levels on the Problem Gambling Severity Index (PGSI) than land-based gamblers (Gainsbury et al., 2014b; Kairouz et al., 2012). In the study by Wu et al. (2015) conducted in Macao, more symptoms of pathological gambling were reported by online gamblers in both selected samples: one representative of the adult population and the other representative of university students. Furthermore, two articles show that the first gambling experience for online players was at a younger age than for land-based players (Wu et al., 2015): approximately 19 years for online players and 24 years for offline players (Dowling et al., 2015), highlighting that an earlier onset of gambling behaviour is more likely to be associated with the online mode (Granero et al., 2020).

Most of the variables reported above are in common with the risk factors for online problem gambling. In fact, problematic gamblers’ behaviour is more likely characterized by greater involvement: high frequency (intensity) (Barrault & Varescon, 2016; Braverman & Shaffer, 2012; Dufour et al., 2013; Gainsbury et al., 2014c; Griffiths et al., 2010; Hing et al., 2017; Hopley & Nicki, 2010; LaPlante et al., 2014; MacKay & Hodgins, 2012; McCormack et al., 2013a; McCormack et al., 2013b), participation in several different gambling forms (high variability) (Braverman & Shaffer, 2012; Gainsbury et al., 2014b, 2015a, 2015c; Hing et al., 2017; LaPlante et al., 2014; Lloyd et al., 2010a, 2010b; McCormack et al., 2013b; Perrot et al., 2018), high expenditure (Barrault & Varescon, 2013b; Barrault & Varescon, 2016; Dufour et al., 2013; Gainsbury et al., 2014b, 2014c, 2015c; Griffiths et al., 2010) and indebtedness (Gainsbury et al., 2012, 2016). In terms of the effects on expenditures, as was assumed in Gainsbury et al. (2015c), compared to nonproblematic or at-risk gamblers, problem gamblers reported a greater amount of money lost through gambling and a greater amount of household debt. An additional gambling behaviour more likely associated with online at-risk gamblers is the longer session duration duration (Barrault & Varescon, 2013a, 2013b, 2016; Griffiths et al., 2010; McCormack et al., 2013b). Although investigated by a few articles, problem gambling risk factors also include early onset of gambling (Granero et al., 2020; Wu et al., 2015), the use of mobile devices compared to computers (Gainsbury et al., 2016), gambling for more than 9 years, not entertaining virtual interactions (Khazaal et al., 2017) and gambling in solitude (McCormack et al., 2013b). Finally, four studies identify being a “mixed-mode” gambler who gambles both online and offline as a risk factor for problem gambling gambling (Dufour et al., 2013; Gainsbury et al., 2015b; MacKay & Hodgins, 2012; Wardle et al., 2011). Mixed-mode gamblers had more symptoms and higher levels of severity than internet-only gamblers. However, this evidence needs further investigation and discussion, since only a minor number of the studies uses the mixed mode method in addition to the dichotomy of online versus offline.

### Risky Behaviours

As reported in previous paragraphs, gambling is often associated with other types of risk behaviour, such as substance misuse. Even though this correlation is valid for all kinds of gamblers, what emerges from review studies is that online gamblers are more likely to use or misuse substances than offline gamblers both while gambling and at other times (Dowling et al., 2015; Gainsbury et al., 2014b; Griffiths et al., 2011; Harris et al., 2013; Kairouz et al., 2012; Mihaylova et al., 2013; Shead et al., 2012; Wood & Williams, 2011). According to Gainsbury et al. (2014b), a significantly higher proportion of internet gamblers report drinking and smoking while engaging in land-based gambling compared to offline gamblers. In contrast, in Goldstein et al. (2016), consuming more substances while gambling was associated with being less likely to be online gamblers. In the internet gamblers group, more people reported hazardous drinking (Dowling et al., 2015; Griffiths et al., 2011), alcohol consumption and addiction (Kairouz et al., 2012; Mihaylova et al., 2013). In relation to the use of other substances, online gamblers are more likely to consume and misuse regular drugs (Dowling et al., 2015), illicit drugs (Mihaylova et al., 2013) and cannabinoids (Dowling et al., 2015; Kairouz et al., 2012). According to Gainsbury et al. (2014b), offline gamblers are more likely to be nonsmokers than online gamblers; concordantly, a significantly higher proportion of online gamblers smoked daily than land-based gamblers.

The relevant use of alcohol, tobacco and drugs represents a risk factor for the development of a problematic online gambling patterns (Gainsbury et al., 2014b; Granero et al., 2020; Lloyd et al., 2010a), even if in some studies, only the number of cigarettes smoked is higher in the riskiest gamblers (Harris et al., 2013; McCormack et al., 2013b). As reported above, it seems that consumption of alcohol or other substances during gambling is more likely associated with online problem gamblers than nonproblem gamblers (Gainsbury et al., 2015c; Harris et al., 2013; Hing et al., 2017; McCormack et al., 2013b).

Risky behaviours related to gambling do not end with excessive substance use; there are other behaviours associated with online and problem gambling, for example, the excessive use of the media. Among the factors that are more likely associated with online gambling are the early use of computers (Hubert & Griffiths, 2018) and being experienced in computer gaming (Edgren et al., 2017). Concordantly, Lelonek-Kuleta et al. (2020) found that people with lower daily internet use are less involved in online gambling. The relevant involvement in gaming was also found to be a risk factor for the development of a problematic gambling pattern (Khazaal et al., 2017).

Deliberate self-harm is another risky behaviour that, according to Lloyd et al. (2010a), is more prevalent among the most problematic cluster of online gamblers (multiactivity players) compared to others.

### Health and Wellbeing

#### Physical Health

Health and well-being are scarcely investigated in the papers included in this review, and their results are almost contradictory. For example, in Wardle et al. (2011), online gamblers were more likely to report that their general health was better than that of land-based gamblers. Regarding physical wellbeing, Shead and colleagues (2012) showed that land-based university student gamblers were more likely to be normal weight, while internet gamblers were more likely to be underweight, overweight, or obese. Furthermore, a physical disability or a significant mental health problem is a predictor of internet gamblers more than offline gamblers (Wood & Williams, 2011). According to Redondo (2015), online gamblers are less interested in their future personal health; in fact, they are more likely to engage in unhealthy activities.

In line with the above, the only risk factor for the development of a pathological mode of gambling emerged from the study by McCormack et al. (2013b) with a sample of online gamblers. Problem gamblers were found to be more likely to report a disability than nonproblem gamblers.

#### Psychological distress and emotions

Regarding psychological well-being, only a few studies have reported significant differences between online and offline gamblers. Gainsbury et al.’s (2014b) paper contends that online gamblers are more likely to experience psychological distress than land-based gamblers. A further relevant result was shown by Goldstein et al. (2016), who monitored the mood of a sample of young adults for 30 consecutive days. The data collected show that those who used the internet to gamble experienced greater negative affect, with higher frequency and intensity, during the observation compared to nononline gamblers.

The high occurrence of cross-sectional studies does not allow us to clearly define the relationship’s direction between psychological distress and problem gambling. It is difficult to establish whether the former is a risk factor or an outcome of the latter. For example, it is unclear whether a high level of psychological distress is a consequence of frequent gambling or conversely whether people with psychological distress are particularly attracted to gambling. Predictably, a higher level of psychological distress was found in online gamblers more at risk of problem gambling than in low-risk gamblers (Gainsbury et al., 2014b; Granero et al., 2020; Hing et al., 2017; Hopley & Nicki, 2010). Anxiety and depression were the main experiences studied and reported by pathological gamblers at higher rates (Barrault & Varescon, 2013a; Barrault et al., 2017; Hopley & Nicki, 2010; Khazaal et al., 2017). In addition, mood disturbances such as hypomanic experiences and mood elevation are reported to a greater extent in the most problematic cluster (Lloyd et al., 2010a). Additional emotional states that are more likely associated with a riskier mode of gambling are dissatisfaction with life (Wu et al., 2015) and feelings of loneliness (Khazaal et al., 2017). Furthermore, problem gamblers and at-risk gamblers were significantly more likely to feel euphoria, excitement, anger, and happiness while gambling (McCormack et al., 2013b). According to the authors, problem gamblers are more likely to experience extreme emotional highs and lows than nonproblem gamblers. In addition, having good emotional intelligence serves as a protective factor against the development of a problematic mode of gambling (Schiavella et al., 2018). A high level of emotional awareness, assertiveness, self-care (understanding and acceptance of self), independence (no emotional dependency), and self-actualization are all aspects that decrease the risk of experiencing a gambling disorder.

### Personality Characteristics and Cognitive Components

#### Personality Characteristics

Regarding personality characteristics, little has been reported for online vs. offline gamblers. According to Redondo (2015), online gamblers are more likely to be characterized by a low degree of sociability and a higher level of frugality.

Variables associated with personality were more relevant in the comparison between those who were at risk of developing problematic gambling. Impulsivity, or the tendency to implement behaviours without considering the possible consequences (Zuckerman & Kuhlman, 2000), is the most widely investigated personality trait and appears to be particularly associated with pathological gambling patterns (Barrault & Varescon, 2013b, 2016; Hopley & Nicki, 2010; Khazaal et al., 2017; Moreau et al., 2020). Other personality traits that increase the likelihood of incurring a problematic mode of gambling are the predisposition to boredom (Hopley & Nicki, 2010) and the lack of premeditation (Khazaal et al., 2017).

In Granero et al. (2020), it was generally found that those who have a dysfunctional personality profile (characterized, for example, by high scores in the novelty-seeking dimension) have a higher likelihood that their gambling will result in a disorder. Conversely, people with functional personality characteristics have a lower likelihood of experiencing problematic gambling. In addition, high scores on the trait of self-direction, which is the ability to adjust behaviour to the demands of the situation to achieve their goals, and in the trait of cooperativeness are considered protective factors associated with adaptive emotional and cognitive responses (Granero et al., 2020).

#### Cognitive Components

Some dysfunctional thinking mechanisms are found to have an influence on the likelihood of being an online gambler. Compared to offline gamblers, internet gamblers are more likely to have cognitive distortions of two main types: the illusion of control and perseverance (Dufour et al., 2020; MacKay & Hodgins, 2012). In Wood and Williams (2011), the illusion of being able to manipulate the outcome of the game has been identified as a risk factor.

The presence of cognitive distortions about gambling increases the likelihood of developing problematic gambling (Barrault & Varescon, 2013a; Gainsbury et al., 2014c, 2015c; MacKay & Hodgins, 2012; Moreau et al., 2020; Schiavella et al., 2018). Comparing low-risk gamblers and those who are pathological, the latter report significantly greater levels in all five types of cognitions analysed in the Gambling Related Cognition Scale (GRCS): gambling-related expectancies, the illusion of control, predictive control, the perceived inability to stop gambling, and interpretative bias. Additional risk factors found in the analysis of poker players and associated with problem gambling include episodes of dissociation while playing (Hopley & Nicki, 2010) and frequent tilt episodes (Moreau et al., 2020).

### Representations, Attitudes and Motivation to Gamble

#### Representations and Attitudes

A gambler's attitude towards gambling has been found to be relevant in influencing the choice of gambling mode. Articles suggest that having a positive attitude towards online gambling increases the likelihood of gambling on the internet (Gainsbury et al., 2012; Harris et al., 2013; Wood & Williams, 2011; Wu et al., 2015). In Gainsbury et al. (2012), internet gamblers experienced higher scores in items investigating the morality, legality, and cost‒benefit of online gambling. In addition to attitudes, a higher level of trust in the internet was more likely to be associated with online gamblers than with offline gamblers (Redondo, 2015). In the article by Harris et al. (2013), a significant difference emerged between the group of online gamblers and the group of land-based gamblers: internet gamblers reported higher scores for the items related to confidence in the security of both online payments and websites than land-based gamblers. Moreover, Redondo (2015) showed that online gamblers have a lower religious orientation than offline gamblers and are less interested in the future of the environment, so they participate less in environmentally responsible activities.

Attitudes towards gambling also appear to influence the likelihood of developing problematic gambling. High-risk internet gamblers are found to have a more negative attitude towards gambling (Harris et al., 2013; Hing et al., 2017). In the article by Gainsbury et al. (2015c), problem gamblers were more likely to believe that the harm of gambling outweighed the benefits, that it was an immoral activity and that all forms of gambling should be illegal. The same result was presented by Hing et al. (2017), who found that problem gamblers reported negative attitudes. This result seems to contrast with findings regarding internet trust, which is associated with a higher likelihood of being problem gamblers (Harris et al., 2013).

#### Motivations to Gamble

Among the motivations that drive a person to gamble, four main reasons are investigated: enhancement, coping, social, and financial. Motivations of enhancement include reasons related to the positive feelings and excitement aroused by gambling; social motivations refer to the willingness to gamble to socialize, spending time with friends or celebrating; coping motivations relate to gambling to relax, to forget problems or because it helps one feel better; and financial motivations refer to the need to get some money, the possibility of winning large sums of money, or wanting to earn money (Lloyd et al., 2010b; Stewart & Zack, 2008). Compared to land-based gamblers, the gambling motivations reported most often by online gamblers are coping reasons (regulating internal state) (Dowling et al., 2015; Goldstein et al., 2016), financial reasons (Barrault & Varescon, 2016) and to satisfy a need for a challenge or to show skills (Dowling et al., 2015; Goldstein et al., 2016). Those who gamble for social reasons (Barrault & Varescon, 2016), because of the positive feelings it elicits (Dowling et al., 2015), or because they believe this activity provides enjoyable social encounters (Goldstein et al., 2016) are more likely to belong to the group of land-based gamblers. Goldstein et al. (2016) analysed the specific motivations for which online gambling is initiated compared to offline gambling. Gamblers were more likely to initiate online activities to win money, to be entertained, or to demonstrate their ability and to discontinue online activities due to feeling bored, tired and distressed. Online gambling activities were less likely to be initiated for social reasons, or because they felt lucky (Goldstein et al., 2016). According to Hubert and Griffiths (2018), comparing online to offline problematic gamblers, the results show that the former are more likely to gamble for fun and leisure.

The same main motivations emerged when investigating online gamblers and comparing them across degrees of severity. Problem gamblers are more likely to report reasons related to the feelings that gambling causes, such as excitement (Gainsbury et al., 2014c), financial aspects (Gainsbury et al., 2014c; Khazaal et al., 2017) or occupational aspects, such as the desire to make money from gambling (Barrault et al., 2017), and coping, as the aim to relax (Khazaal et al., 2017). In contrast to what was previously stated regarding the possibility that coping motivations act as a risk factor, in the article by Gainsbury et al. (2014c), it appears that gambling to relax is reported more by nonproblem gamblers. In addition, nonproblem gambling appears to be associated most often with leisure and coping purposes, such as for pleasure, experiencing positive emotions, a distraction from everyday life and thus relaxation (Barrault & Varescon, 2013b, 2016) and as an occasion of social gathering (Khazaal et al., 2017).

The aspects associated with the intrinsic characteristics of online gambling, which were discussed in depth in the introduction, are investigated to a lesser extent in this review’s papers. Compared to offline gamblers, online gamblers report greater motivation due to accessibility, availability, variability in sites and activities, anonymity, and prevention/protection (Hubert & Griffiths, 2018). In addition, greater accessibility and anonymity are two of the reasons more likely to be reported by problem gamblers than by nonproblem gamblers (McCormack et al., 2013b).

### Risk and Protective Relational and Contextual Factors

#### Relational Factors

The choice of gambling modality appears to also be influenced by aspects related to the network of the gambler’s relationships, even if they are poorly investigated in comparison to individual factors. Studies show that low quantity and quality of the relationships of those who gamble play a role in increasing the likelihood of being internet gamblers. An additional factor associated with the online mode is reporting the subjective presence of issues within the household due to gambling (Mihaylova et al., 2013). At the relational level, a single factor has been identified that increases the likelihood of developing problematic gambling: the presence of gamblers and problem gamblers among family members. This result has been reported by two different authors who considered the general adult population (Lloyd et al., 2010a) and university students (Harris et al., 2013).

#### Contextual Factors

The surroundings and life contexts to which a person belongs play an important role in influencing gambling, as do individual and relational factors. Within the selected articles, variables acting at the contextual level were scarcely investigated. The university context, among all, is the only setting that has been investigated and for which there is evidence of a risk factor. The presence of academic issues in the population of university students appears to increase the likelihood that not only they will use the internet to gamble (Mihaylova et al., 2013), but also they will become problem gamblers (Harris et al., 2013).

## Discussion

This paper provides a synthesis of knowledge regarding the risk and protective factors of online gambling in the adult population. From the analysis carried out, several critical elements emerge, which may offer indications for future studies. Regarding the methodology used in the studies, two critical issues emerge concerning the population and the method. Most of the papers use nonrepresentative samples. For future research, it would be desirable to use representative samples of the population. In addition, most of the papers are cross-sectional studies, whereas it would be desirable to conduct longitudinal studies to achieve a greater understanding of the relationship between variables. It is necessary to highlight that in most papers, the sample was mainly composed of men, no women. Studies that included women reported that these gamblers were at greater risk of developing problematic gambling and were more attracted to internet gambling. This topic was explored in a qualitative study by Corney and David (2010) that focuses on the motivations of female online gamblers. This article suggests that aspects related to ease of access and anonymity of gambling are particularly relevant for women. In fact, the possibility of gambling from home and remaining anonymous make online gambling more attractive to women, as they perceive it to be safer and less intimidating. For these reasons, it would be relevant in future research to use a representative sample.

Several factors were identified in the review. Socioanagraphic variables are among the most studied in both comparisons. Gender, age, level of education, occupation, income and marital status are largely investigated. Being male and younger seem to be associated more with online gamblers than offline gamblers and with problematic online gamblers than nonproblematic gamblers. Moreover, a high level of education, income, and job status are more likely associated with online gamblers than offline gamblers. At the same time, looking at online gamblers, it seems that these factors are more related to less problematic gamblers than problematic gamblers. Other contradictory results regard marital status or the sentimental relationship. It seems that having a stable partner is more likely associated with online gambling than offline gambling, even though it is more associated with nonproblem gamblers than problem gamblers. Having dependent children is more likely associated with online and problematic gamblers, but it is studied by only a few papers.

Gambling patterns and behaviors is the second most studied factors category. A relevant number of papers show that high intensity, high variability, and high expenditures in gambling are more likely associated with online gamblers and represent risk factors for problematic gambling. The same association is reported concerning long session duration and having an early onset of gambling behaviour. Some factors are studied only for the second comparison. Among these, solitary gambling (not using virtual chats or forums), being a mixed-mode and long-time gambler, using mobile devices to gamble, and having tilt episodes represent risk factors for problem online gamblers, even though only a few studies show these results.

Risky behaviours, such as the consumption of alcohol, drugs, and tobacco, are studied in both comparisons. The misuse of substances is more likely associated with online gamblers than offline gamblers and with online problematic gamblers than less problematic gamblers. Moreover, the same association is reported for high use of media, while deliberate self-harm is more likely to be found among problem online gamblers.

Factors related to physical well-being are poorly investigated, and mainly concern the comparison between online and offline gamblers. It seems that offline gamblers are more interested in engaging in healthy activities, are fitter and generally feel healthier than online gamblers.

Psychological dimensions are slightly investigated, and most of these papers study only the second comparison. Online problem gamblers are more likely to report psychological distress and anxious or depressive states than nonproblem gamblers. A smaller number of studies reported that negative moods, extreme emotions while gambling, and mood disturbance are more likely associated with online problem gamblers. However, one paper shows how high emotional intelligence (emotional awareness, assertiveness, self-care, independence, self-actualization) could act as a protective factor, but further investigation is needed.

Personality traits have not been extensively explored. High impulsivity is the most often studied factor, and it is associated most often with online problematic gamblers as much as having a dysfunctional personality. In contrast, online gamblers compared to offline gamblers seem to have a minor degree of sociability and a higher level of frugality, but it is only stated by a single paper. Concerning the cognitive components, the abundant presence of cognitive distortion in gambling (as the illusion of control) is more likely associated with online and online problematic gamblers than with offline and nonproblematic gamblers.

Attitude towards gambling has been found to be relevant in influencing the choice of gambling mode. Articles suggest that having a positive attitude towards online gambling is more likely associated with internet gambling, while a negative attitude is related more often with problem gambling. This result should be further investigated.

Among the different reasons to gamble, social motivations are more often related to offline and nonproblem gamblers, while financial reasons are more often associated with online and problem gambling. Contradictory results emerged regarding coping and pleasure reasons, and it is not clear how these motivations influence gambling behaviour, so further studies will be needed.

Scarce attention is given to relational factors and contextual factors. A few papers suggest that having rare and negative relationships is more likely associated with online gambling. Moreover, having family members who gamble could influence the likelihood of being a problem gambler. In addition, having problems in life contexts such as academia is reported mainly by people who gamble online and are problematic gamblers.

The results of the review regarding risk and protective factors show that risk factors are investigated to a greater extent than protective factors. This criticality highlights the need to strengthen research from a well-being-promotion approach to identify and then intervene on variables related to positive outcomes. In addition, among the levels of analysis studied in the literature, the most in-depth level concerns individual aspects, while both the relational and contextual levels are poorly investigated. Future research would need to embrace a psychosocial perspective that considers, at least equally, all types of levels, valuing the influence that the environment has on the individual. Moreover, some of the factors’ categories are scarcely investigated in the literature; for this reason, they need to be explored in greater depth. Examples include variables associated with physical well-being, emotional and social functioning, and interpersonal skills. One of the recurring themes among the categories concerns bonding with other people. In general, it appears that the presence of other people in different contexts of life acts as a protective factor for problematic gambling, while the absence of these represents a risk factor. Although the relational level is poorly investigated within the review, the positive influence of relationships is studied at the individual level. For example, being married or being in a relationship with a stable partner, and among the factors associated with gambling patterns, playing while in the company of others represents a protective factor. Similarly, sociality is also present in motivational aspects, and those who gamble to meet other people, celebrate, and be with friends are less likely to be problem gamblers. These results refer to the importance that the social sphere has on the individual, which is essential. This theme needs to be studied to a greater extent and to be taken into consideration from the point of view of intervention and prevention.

Most of the factor results are in line with what emerged from Gainsbury’s review (2015) and previous literature about risk factors for problem gambling. For example, several risk factors for problem gambling were confirmed: being male, being a young adult, having gambling behaviours characterized by high intensity, variability and high expenditures, gambling for long periods of time, having an early onset of gambling behaviour, misusing substances, and reporting psychological distress, impulsivity, and cognitive distortions related to gambling. Moreover, having academic and familiar issues or familiarity with gambling are risk factors for problem gambling. However, many other protective and risk factors emerged from this review, such as social support, healthy lifestyle, emotions, motivations and technology use and interactions with others. This review differs from Gainsbury's in that it attempts to use an additional and more systematized classification to the reading of risk and protective factors of gambling. Specifically, the papers included in the review are classified depending on two different comparisons: according to the degree of severity of online gambling and the differences between online and offline gambling. Including these two comparisons is crucial to account for the complexity of online gambling and the different targets involved. Analogies and differences emerged from these two comparisons, and specific needs of further investigations have been identified. For example, contradictory results emerged about gender differences, level of education influence, emotional skills, attitudes and motivational issues.

In conclusion, aiming to fill the literature gap on preventive factors for online gambling, the results of this literature review can provide the basis for developing efficient preventive strategies that go beyond responsible gambling options offered by gambling platforms (Gainsbury et al., 2014a; Velasco et al., 2021). These findings contribute to identifying the groups most attracted to online gambling and most vulnerable to the development of problem gambling. These people should be the focus of future research and targeted individualized interventions. From a more general prevention perspective, more coordination between research evidence, agencies, and institutions is needed to support policies and a social culture unfavourable to gambling to protect the health of online gamblers. Specifically, given the commonalities between risk and protective factors for online and offline gambling, it does not seem necessary to create new prevention interventions dedicated directly to online gambling. On the one hand, given the presence of aspects related only to online gamblers and given the differences in terms of socioanagraphic variables, it would seem to make sense to reevaluate some of the interventions to adapt them to these specificities. For example, given that even gamblers from populations considered less at risk (highly educated and employed) seem to be highly attracted to gambling, it would be important to target them with specific interventions or include them in a universal intervention. On the other hand, it appears that gamblers with fewer resources are more likely to become problematic gamblers and thus would need to be involved in indicated interventions to promote or enhance protective factors. More attention should be given to acknowledging and dealing with the taboo of female gamblers; despite being an extremely valuable topic, it was not covered much by the articles included in the review. Finally, the relevance of social relationships and sociality during gambling should be considered when designing online gambling preventive interventions. Online access to gambling facilitates solitary play and isolating habits, and social protective factors could be reduced.

### Limitations of the Review

This review presents some limitations. No statistical processing typical of meta-analyses to assess the results has been included. However, the ability of this review to synthesize the evidence across a large body of literature offers a valid overview and some recommendations. Regarding the included studies, not all papers displayed the same level of methodological quality, and the criteria used for the studied population were quite different. Moreover, the literature lacks a clear and determined definition to distinguish online and offline gamblers. In fact, some authors consider that only those who exclusively use this mode are online gamblers, while others define them as such even if they mainly use the online mode but also gamble offline. Given the heterogeneity of the literature and the need to synthesize and systematize the results, the information regarding “exclusively internet gamblers” or “mixed mode gamblers” was included in the same “online gamblers” category regardless of the definition used by the authors. The reason behind this choice is that this distinction of exclusivity was made explicit only in a few papers, so we considered online gamblers who play at least partially online. Moreover, because there is no univocal and agreed definition to classify online gamblers depending on the intensity of gambling, we considered the category online gamblers without distinguishing the different definitions of the authors. For example, some authors consider online gamblers to be those who gamble at least once a year, others if the frequency is once a month, they were both just addressed as “online gamblers”. Furthermore, in the literature, there is no clear and shared definition and categorization of gamblers depending on the degree of severity of problem gambling. Some authors distinguish between low-, medium-, and high-risk gamblers, while others consider only nonproblem or problem gamblers. To synthesize, the results of the papers are read without valuing the intermediate degrees of risk, distinguishing only between problematic or nonproblematic gambling. Finally, given that only some papers considered only specific subpopulations of gamblers (e.g., poker players, sports bettors), the results of the papers were considered net of gambling types.

## Conclusion

The aim of this paper was to review the knowledge and evidence about the factors that influence the likelihood of being an online gambler and developing a problematic mode of gambling in the adult population. The review synthesized and systematized the risk and protective factors associated with online gambling. Specifically, to do so, two types of comparisons were made: comparison of factors that distinguish offline from online gamblers and comparison of online nonproblematic gamblers from online problematic gamblers. In addition, a further comparison was carried out to highlight whether similarities or differences emerged in the results with respect to the factors studied between the first and second comparisons. The results of this work could be useful in suggesting directions for the development of prevention programs targeted at offline and online gamblers, which could be aimed at strengthening or increasing protective factors and limiting and reducing risk factors. Moreover, this review provides some suggestions for distinguishing characteristics more associated with online problem gambling and non-problem ones. Finally, this review found that even if most risk and protective factors are in common between online and offline gamblers, there are some variables that are not. These factors could be important to consider in project prevention interventions aimed at targeted online gamblers and online problematic gamblers.

## Data Availability

Data sharing is not applicable to this article as no new data were created or analysed in this study.

## Appendix A: Systematization of the Review’s Results

**Table:**

Category	Factors	Description	N° Papers	(C1) Online (ON) vs Offline gamblers (OFF)	(C2) Online Non problem (ON-NP) vs Online problem gamblers (ON-P)	Consistency of results C3
Sociodemographic information	Sex	ON and ON-P are more likely to be male and younger	20	3, 10, 12, 15, 16, 18, 24, 27, 32, 33, 34, 35, 37, 38, 39	9, 11, 16, 21, 22, 25	Almost complete consistency across comparisons
	Age		16	3, 5, 10, 15, 16, 18, 26, 32, 37, 38, 39	4, 9, 11, 20, 22, 26,	
	Level of education	ON are more likely to have a higher level of education; ON-P are more likely to have a lower level of education	8	15, 16, 18, 19, 37, 39	6, 20	Different results in the two comparisons
	Income/ socioeconomic status	ON are more likely to have a higher income; ON-P are more likely to have a lower income	9	3, 10, 16, 18, 33, 37, 38,	4, 11	
	Occupation	ON are more likely to be full-time employed with higher job status; ON-P are more likely to be unemployed or with lower job status	12	5, 10, 18, 26, 33, 37, 38, 39	4, 8, 20, 22	
	Marital status/relationship	An equal number of articles state that both being in a stable/married relationship and being single/unmarried are associated with ON. In contrast, being single/unmarried is a risk factor for ON-P. Being married/being in a stable relationship decreases the likelihood of being ON and ON-P	10	5, 18, 32, 33, 37, 38, 39	4, 9, 20	Mixed results for risk factors in online vs offline comparisons.
	Dependent children	Having dependent children is associated with ON and acts as a risk factor for ON-P.	3	5, 18	3	Common results in the two comparisons
	Place of residence	Living in the country and living in a metropolitan area are both associated with ON.	2	3, 19	/	/

**Table:**

Category	Factors	Description	N°	C1 ON vs OFF	C2 ON-NP vs ON-P	Consistency of results C3
Gambling pattern and behaviour	Degree of severity	ON are more likely to report a higher degree of gambling severity	**11**	1, 12, 16, 21, 24, 31, 32, 34, 37, 38, 39	/	/
	Intensity	ON and ON-P are more likely to gamble at higher intensity and on more types and forms of gambling	**19**	5, 12, 13, 18, 26, 27, 31, 33, 32, 33, 34, 35	11, 13, 22, 23, 25, 29, 31, 34, 40, 41, 42	Common results in the two comparisons
	Variability		**21**	1, 10, 18, 26, 27, 32, 33, 34, 35, 37, 38	7, 11, 17, 20, 21, 23, 25, 26, 29, 36, 41	
	Expenditure	ON and ON-P both are more likely to report higher gambling expenditure and indebtedness	**12**	12, 13, 18, 27, 31, 32, 38,	13, 20, 21, 22, 28, 31, 42	Common results in the two comparisons
	Indebtedness		**4**	27, 38	9,20	
	Session duration	ON and ON-P are more likely to gamble for longer session and have had an early onset	7	13, 32	13, 22, 25, 28, 30, 42	Common results in the two comparisons
	Early onset		**3**	16, 18	4, 16	
	Being a long-time gambler	ON-P are more likely to be a long time gambler and to gamble alone	1	/	25	/
	Gambling alone/with someone		2	/	9, 25	/
	Virtual interactions (Chat/forum)	ON-NP are more likely to engage in virtual interactions	1	**/**	9	**/**
	Mode and device of access	ON-P are more likely using mobile devices	1	**/**	14	**/**
	Tilt episode	ON-P are more likely to have tilt episode	1	**/**	2	**/**
	Mixed - mode	ON and ON-P are more likely to gamble online and offline	4	34	19, 31, 37

**Table:**

Category	Factors	Description	N°	C1 ON vs OFF	C2 ON-NP vs ON-P	Consistency of results C3
Risky behaviours and addictions	Substance use while gambling	ON and ON-P are more likely to use substance while gambling	6	12, 21	11, 20, 24, 25	Common results in the two comparisons
	Alcohol and drug use	ON and ON-P are more likely to misuse substances as alcohol, drugs and tobacco	11	18, 21, 24, 27, 32, 33, 35, 38, 39	4, 21, 41	
	Tobacco		8	21, 24, 35, 38, 39	4, 25, 41	
	Internet and computer	ON and ON-P are more likely to use excessively the internet and computer	4	3, 5, 10	9	
	Deliberate self harm	ON-P are more likely to report self harm	1	/	41	/
Physical health	General Health status	ON are more likely to report a worse general health status, the presence of disability, an unhealthy weight and life style	1	37	/	Few studies are available, this level of analysis requires further investigation.
	Disability		3	37, 38	25	
	BMI		1	35	/	
	Unhealty activities		1	15	/	
Psychological distress and emotions	Psychological distress mental health problems	ON e ON-P are more likely to report higher level of psychological distress	5	21, 38	4,11, 21, 40	Common results
	Anxiety and depression	ON-P are more likely to report high level of anxiety and depression	6	/	2,8,9,30, 31, 40	/
	Negative mood state	ON are more likely to report negative mood state	1	12	/	/
	Loneliness and dissatisfaction	ON-P are more likely to report high level of loneliness and dissatisfaction in life, to feel extreme emotions while gambling and to report mood disturbance	3	/	9, 16, 25	/
	Extreme emotion while gambling		1	/	25	/
	Mood disturbance		1	/	41	/
	Emotional intelligence	ON-NP are more likely to report higher level of emotional intelligence	1	/	6	Requires further investigation

**Table:**

Category	Factors	Description	N°	C1 ON vs OFF	C2 ON-NP vs ON-P	Consistency of results (C3)
Personality characteristics	Impulsivity	ON-P are more likely to be more impulsive	5	/	2, 9, 13, 28, 40	/
	Dysfunctional personality		1	/	4	/
	Degree of sociability	Common results in the two comparisons	1	15	/	/
	Frugality; Boredom proneness		2	15	40	/
Attitudes and cognitive components	Cognitive Distortions	ON and ON-P are more likely to report more cognitive distortions about gambling	10	1, 31, 34, 38	2, 6, 9, 20, 22, 30, 34	Common results in the two comparisons
	Attitudes toward online gambling		6	16, 24, 33, 38	11, 20, 24	Conflicting results
	Trust in internet	ON and ON-P are more likely to trust internet more	**2**	15, 24	24	Common results in the two comparisons
	Religious orientation; Environmental responsibility	ON and ON-P are more likely to report low level of religious orientation and environmental attention	**1**	15	**/**	**/**
Motivations	Financial reasons	ON and ON-P are more likely to gamble for financial reasons	5	13	9, 22, 25	Common results in the two comparisons
	Coping; Relaxation reasons	ON are more likely to gamble for coping and pleasure reasons, contrasting results in C2	**5**	12, 18	9, 22, 25	Conflicting results
	Pleasure/Excitement reasons		6	5, 12, 18	13, 22, 28	
	Social reasons	OFF and ON-NP are more likely to gamble for social reasons	3	12, 13	9	Common results in the two comparisons
	Challenge/skills reasons	ON are more likely to gamble for challenge or show skills	2	12, 18	/	/
	Peculiarities of online gambling	ON and ON-P are more likely to gamble for the characteristics of online gambling	2	5	25	Common results in the two comparisons

**Table:**

Category	Factors	Description	N°	C1 ON vs OFF	C2 ON-NP vs ON-P	Consistency of results (C3)
Relational and contextual factors	Family problems	ON are more likely to have problematic family situation	1	27	**/**	This level of analysis need further investigation
	Gambling issue among relatives	ON-P are more likely to report relatives with gambling problem	2	/	24, 41	
	Academic problems	ON and ON-P are more likely to report academic difficulties	2	27	24	Common results

## Appendix B

List of the articles included in the review

**Table:**

N°	Authors	Year of pub.
1	Dufour et al.	2020
2	Moreau et al.	2020
3	Lelonek-Kuleta et al.	2020
4	Granero et al.	2020
5	Hubert & Griffiths	2018
6	Schiavella et al.	2018
7	Perrot et al.	2018
8	Barrault et al.	2017
9	Khazaal et al.	2017
10	Edgren et al.	2017
11	Hing et al.	2017
12	Goldstein et al.	2016
13	Barrault & Vareson	2016
14	Gainsbury et al.	2016
15	Redondo	2015
16	Wu et al.	2015
17	Gainsbury et al.	2015a
18	Dowling et al.	2015
19	Gainsbury et al.	2015b
20	Gainsbury et al.	2015c
21	Gainsbury et al.	2014b
22	Gainsbury et al.	2014c
23	LaPlante et al.	2014
24	Harris et al.	2013
25	McCormack et al.	2013b
26	Gainsbury et al.	2013
27	Mihaylova et al.	2013
28	Barrault & Varescon	2013
29	McCormack et al.	2013a
30	Barrault & Varescon	2013
31	Dufour et al.	2013
32	Kairouz et al.	2012
33	Gainsbury et al.	2012
34	MacKay & Hodgins	2012
35	Shead et al.	2012
36	Braverman & Shaffer	2012
37	Wardle et al.	2011
38	Wood & Williams	2011
39	Griffiths et al.	2011
40	Hopley & Nicki	2010
41	Lloyd et al.	2010
42	Griffiths et al.	2010
